# Ultrasound Imaging of Crural Fascia and Epimysial Fascia Thicknesses in Basketball Players with Previous Ankle Sprains Versus Healthy Subjects

**DOI:** 10.3390/diagnostics11020177

**Published:** 2021-01-26

**Authors:** Carmelo Pirri, Caterina Fede, Antonio Stecco, Diego Guidolin, Chenglei Fan, Raffaele De Caro, Carla Stecco

**Affiliations:** 1 Department of Neurosciences, Institute of Human Anatomy, University of Padua, 35121 Padua, Italy; caterina.fede@unipd.it (C.F.); diego.guidolin@unipd.it (D.G.); yutianfan1218@163.com (C.F.); rdecaro@unipd.it (R.D.C.); carla.stecco@unipd.it (C.S.); 2 RUSK Rehabilitation, New York University School of Medicine, New York, NY 10016, USA; antonio.stecco@nyulangone.org

**Keywords:** connective tissue, fascia, basketball sport, Muscle-skeletal Ultrasound, Y-balance test, ankle sprain

## Abstract

Background: Fascial layers may play an important role in locomotor mechanics. Recent researches have revealed an association between increases of fascia thickness and reduced joint flexibility in patients with chronic pain. The purpose of this study was to measure and compare, through the use of ultrasound imaging, the thickness of the deep/crural fascia in different points of the leg as well as the epimysial fascia thickness at level 2 of anterior compartment of leg, in male basketball players with history of recurrent ankle sprain and in healthy participants. Methods: A cross-sectional study has been performed using ultrasound imaging to measure deep/crural fascia thickness of anterior, lateral and posterior compartment of the leg at different levels with a new protocol in a sample of 30 subjects, 15 basketball players and 15 healthy participants. Results: Findings of fascial thickness revealed statistically significant differences (*p* < 0.01) in epimysial fascia thickness and in deep/crural fascia thickness between levels/compartments of the same group and between two groups. Moreover, Post 3 deep/crural fascia thicknesses (*p* < 0.001) were decreased showing statistically significant difference for the basketball players group respect the healthy participants group. Conclusions: These findings suggested that the posterior compartment was thicker than anterior compartment, probably due to a postural reason in both groups. Moreover, they showed an increase of thickness of the epimysial fascia in basketball players with previous ankle sprains. This variability underlines the importance to assess the fasciae and to make results comparable.

## 1. Introduction

Various studies investigated the value of Ultrasound (US) examination of fasciae [1,2,3,4,5] and demonstrated efficient assessment of the deep fasciae and measurement of the thickness of the subcutaneous and perimuscular connective tissue more economic than other non-invasive methods [4,6]. Langevin and colleagues also investigated gliding between the muscles and adjacent fascial layers and between the various fascial layers [1]. According to some studies, there is a good inter-rater reliability in the US fascial assessment [7].

Ankle sprains are one of the most common injuries caused by physical activity during sports or activities of daily living [8,9,10].

They usually happen during dynamic movement, and over 50% of all ankle injuries determine a ligament injury [11]. Chronic ankle instability (CAI) is termed as a subjective feeling of the ankle sagging, due to a scheme of instability with an initial ankle sprain followed by repeated ankle sprains [10]. CAI can be defined as the state caused by the experience of multiple ankle sprains, with instability resulting from limited joint range of motion (ROM) and movement [11].

Additionally, ankle stability can have a positive effect on standing balance and even affect the stability of movements such as walking and jumping [12].

CAI can mostly come about due to an inappropriate treatment following an initial injury and pain in the lateral part of the ankle joint [10,13,14]. The instability and the discomfort during sudden changes of direction or stopping actions, determine in patients with CAI a reduced balance control. Therefore, when ankle injury occurs, the mechanical receptors in the joint become damaged, leading to functional instability [15]. Although ligament damage, reduced muscle strength, delayed muscle response time, and proprioceptive deficits in the ankle are the components of the functional ankle stability, its precise mechanisms have not been elucidated [16,17,18]. In the CAI, dynamic movement is limited, and balance is damaged [19].

Range of motion (ROM), the maximum distance a joint can move, is a significant feature of musculoskeletal health, and international guidelines support regular exercise to preserve or recondition the physiology [19,20,21].

However, the determinants of ROM and their relative contributions have not been fully clarified despite the numerous training approaches. On the one hand, self-perceived stretch tolerance and associated sensations generated by the central nervous system appear as a crucial factor [22]. On the other hand, it has been debated that the flexibility of the soft tissue has an important role [23].

Wilke J et al. [4] explained how the connective tissue and in particular the deep fascia can impact the ROM in various manner [15], indicating that fascia thickness differs in different ages showing regional specializations. Based on thickness and relationship with underlying muscles, two principal types of deep muscular fasciae can be distinguished, the aponeurotic fasciae and the epimysial fasciae. Aponeurotic fasciae refer to all the well-defined fibrous sheaths that cover, and maintain in position, a group of muscles or provide the insertion for a broad muscle, while the epimysial fasciae are specific for each muscle and determine their shape and volume [5].

Y-balance test (YBT) is a common and reliable method for clinical assessment of dynamic balance [24,25,26]. Its scores are known to vary by age, sex and sport [24]. Ankle injuries have been linked to a reduced reach distance in the Postero-medial direction in recreationally active college students [27,28] while ankle sprains in high school and college football athletes were linked to a reduced reach distance in the anterior direction [29].

Numerous studies by Ultrasound imaging have related atrophy of muscles of leg, tendon ruptures, tendinopathies, delayed reaction times of the peroneus muscles, their cross-sectional area, to ankle sprains [30,31,32]. Nevertheless, to date, no study has compared the fascial thicknesses measured by ultrasound imaging in relation to recurrent ankle sprains and chronic ankle instability.

The purpose of the current cross-sectional study was to investigate the difference of the deep/crural fascia thickness in different points of the leg and epimysial fascia thickness in a point of the leg where the best visibility of the structure is possible, among a healthy control group and a group of basketball players with recurrent ankle sprains, whose risk of injuries was confirmed by confirmed by a Y balance test value. The second aim was to assess the correlation between deep/crural and epimysial fascia US thicknesses and flexibility/dynamic balance in basketball players with chronic ankle sprains. We hypothesized that thickness differences could be revealed for the fascial US thicknesses between two groups.

## 2. Materials and Methods

### 2.1. Study Design

A cross-sectional study based on the Strengthening the Reporting of Observational studies in Epidemiology (STROBE) statement was conducted [33] in order to compare the US thicknesses of crural fascia in different compartment and levels of leg and epimysial fascia in the level 2 of the anterior compartment between basketball players with previous ankle’ sprain and healthy participants. The Helsinki Declaration and human experimentation rules [34] were considered and previously, the Ethics Committee of University of Padua approved the research. All participants were informed prior to inclusion in the project by providing a written consent form.

### 2.2. Participants

A total sample of 30 subjects were recruited and divided into two groups: “A” group comprised male basketball players with previous recurrent ankle sprain (*n* = 15) and “B” (control) group of Healthy participants (*n* = 15) all physically active but without history of ankle sprain or trauma to the lower limbs in the past. For “A” group, participants were included if they presented: history of recurrent ankle sprains but absence of them over the previous 6 months with verification of the ankle’ ligaments integrity by physical examination and ultrasound imaging, functional ankle instability shown by abnormal function characterized by recurrent episodes of ankle giving way and positive Y balance test for risk of ankle instability [28].

The exclusion criteria were any lower extremity (e.g., previous fractures, tendinopathies, participants with conditions involving the peroneus quartus muscle, tendon ruptures, neuropathy injuries; past diagnosis of a neuromusculoskeletal condition of the foot or forefoot, e.g., use of plantar orthoses, pes planus and cavus, hallux valgus and rigidus, plantar fasciitis, heel spurs, Morton neuroma, Sever disease, tarsal tunnel syndrome, or entrapment of the deep or superficial peroneal nerve; past diagnosis of a neuromusculoskeletal condition of the leg, e.g., fibular tunnel syndrome or degeneration or inflammation of the tibial periosteum) or back injury, or surgery, severe orthopedic, neuronal, psychiatric, cardiopulmonary, endocrine diseases, under 18 years old, pregnant, with a chronic skin condition (eczema, psoriasis, etc.), previous severe trauma in the inferior limbs, collagen disorder (scleroderma, mixed connective tissue disorder, etc.), and/or chronic medical condition requiring intake of medications. The “B” (control) group was composed of participants with no history of ankle sprains.

The enrollment of the subjects was performed by a specialized medical doctor with more than 5 years of experience in physical and rehabilitation medicine.

### 2.3. Ultrasonography Imaging Measurements

Using a high-resolution device (Edge II, Sonosite) with a frequency range of 6–15 MHz and a screen resolution of 1680 × 1050 pixels, ultrasound images were taken at leg with a specific US scans protocol. A physician specialist in Physical and Rehabilitation Medicine with 5 years’ experience in skeletal-muscle US imaging and US imaging of fasciae carried out the US assessments. A standardized protocol was created and used to assess the fascial layers (deep fascia/crural fascia and epimysial fascia in anterior compartment) for bilateral assessment. The US system speed of sound was c = 1540 m/s, conventionally used in diagnostic US system. The US was set to B-mode and depicted a depth of 30 mm. For adequate scans and to reduce surface pressure on the skin, the ultrasonographer used suitable amounts of gel. The probe was placed on the skin as lightly as possible to avoid tissue compression but quite stable to maintain adequate contact between the probe and skin for consistent images. To eliminate the influence of possible thickness variations, three equidistant region of interest per image/levels for layers were measured; in each of them, three points representing the best visibility for each fascial layer were measured and the resulting values were averaged for analysis. The investigator followed the same protocol to ensure that each point of crural fascia of calf and epimysial fascia (level 2 anterior) was quantified in the same way.

The US beam was kept perpendicular to the fascial layers because anisotropy artifacts typically affect them. The power and overall gain of the ultrasound machine were adjusted to optimize visualization of the fascial planes and obtain the best scans possible [35]. The US images were frozen and captured.

**Figure 1:**
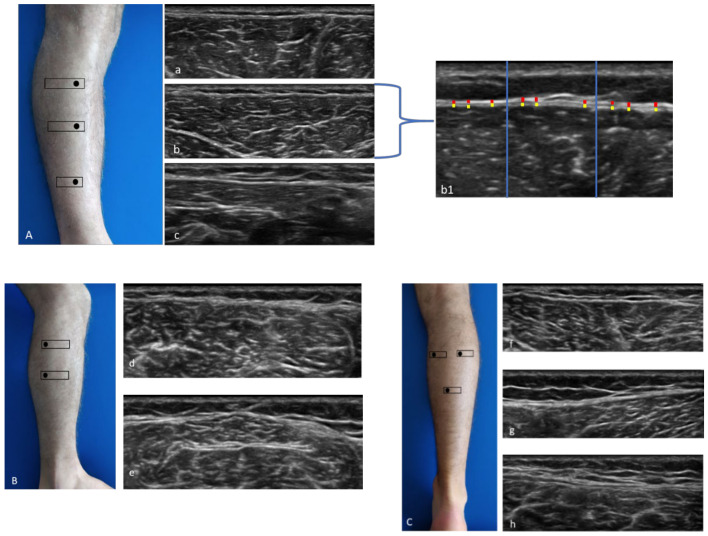
Ultrasound (US) images of the anterior (**A**), lateral (**B**), and posterior (**C**) compartments. Anterior compartment (**A**) at the levels Ant 1 (**a**), Ant 2 (**b**), and Ant 3 (**c**); method of measurement (**b1**). Lateral compartment (**B**) at levels Lat 1 (**d**) and Lat 2 (**e**). Posterior compartment (**C**) at the levels Post 1 (**f**), Post 2 (**g**), and Post 3 (**h**). Probe: black rectangle. Yellow dashes: epimysial fascia; red dashes: crural fascia.

The ultrasonographer used the short axis because is the best to visualize and follow the landmarks correlated with the fascial layers and to have less spatial anisotropy [36]. For each point, following the description of the fascial layers visualization in ultrasound imaging used by Pirri et al. [5], a specific protocol was defined:

1. Anterior region (Figure 1A): Patient relaxed in supine position with right lower limb in neutral position.

Anterior 1 (Ant 1): below the tibial crest, anterior proximal third of the leg (Figure 1A(a)).

Anterior 2 (Ant 2): the probe was moved downwards following the anterior tibialis muscle at level of the anterior middle third of the leg, moving the probe laterally until the muscle belly of the anterior tibialis muscle is centered in the monitor (Figure 1A(b)).

Anterior 3 (Ant 3): the probe was moved downwards, and the image was evaluated at the point before of the superior retinaculum of the flexors (Figure 1A(c)).

2. Lateral region (Figure 1B): Patient in left lateral decubitus to assess right limb and vice versa.

Lateral 1 (Lat 1): the probe was placed transversally to the lateral proximal third of the leg, taking the peroneal head as landmark, above the peroneal longus muscle (Figure 1B(d)).

Lateral 2 (Lat 2): the probe was moved downwards following the peroneal bone until visualization of the peroneal longus muscle with the peroneal brevis muscle underneath (Figure 1B(e)).

3. Posterior region (Figure 1C): Patient prone with lower limb in neutral position.

Posterior 1 (Post 1): the probe was placed axially at the level of medial belly of gastrocnemius muscle under the popliteal fold, maintaining the sural nerve at the center of the image (Figure 1C(f)).

Posterior 2 (Post 2): the probe was moved laterally until the level of lateral belly of gastrocnemius muscle (Figure 1C(g)).

Posterior 3 (Post 3): the probe was moved downwards until that following as landmark the visualization of the sural nerve and the sural vein, visualizing the two bellies of the gastrocnemius muscle and the soleus muscle (Figure 1C(h)).

All measurements for the epimysial fascia were performed only at anterior 2 level, because it was the only area where was possible to the distinction between deep fascia and epimysial fascia of the tibialis anterior muscle.

The images for each region/level were frozen and captured at the end of each assessment, and fascial thickness was measured by Image J analysis software. Each image was divided into three regions; in each of them, three points representing the best visibility were measured and averaged (Figure 1A(b1)). To eliminate the influence of possible thickness variations, three equidistant points per image were measured and the resulting values were averaged for analysis. The scanner settings were kept constant during the study [37].

### 2.4. Statistical Analysis

Statistical analysis was performed using GraphPad PRISM 8.4.2 (GraphPad Software Inc., San Diego, CA, USA), and *p* < 0.05 was always considered as the limit for statistical significance.

The normality assessment was carried out using the Kolgomorov–Smirnov test. Descriptive statistics were calculated for both groups separately, including measures of central tendency and their dispersion ranges using the mean and standard deviation (SD) to describe parametric data. Finally, a comparative analysis between the Basketball players and the healthy group was made performing Student’s t-test. Differences US-estimated thickness across regions/levels were statistically analyzed by one-way analysis of variance (ANOVA) followed by Bonferroni’s test for multiple comparisons. The resulting effect size was calculated by G Power 3.1 according to Cohen’s d and interpretated as small (d = 0.20), medium (d = 0.50) and large (d = 0.80) [38]. For epimysial fascia the effect size was d = 1.22 in a first our pilot study, α err prob = 0.05, power: 1-β err prob = 0.80; sample size was for group = 10 [38]. Nevertheless, we could include a sample of 15 individuals for group. In addition, the Pearson’s test was employed for both groups to evaluate the correlation between BMI, weight, height, age and deep/crural or epimysial fascia; moreover, it was employed for basketball players group to evaluate the correlation between findings of Y-balance test, ROM of ankle and deep/crural or epimysial fascia. Two-way mixed model intra-class correlation coefficient (ICC_3,k_), type C, was used to evaluate the intra-rater reliability. ICC values were interpreted as poor when below 0.5, as moderate when between 0.5 and 0.75, as good when between 0.75 and 0.90, and as excellent when above 0.90 [39]. 95% confidence intervals (CI) are reported parenthetically after the group estimator where applicable. SPSS version 21 was used for all statistical analyses was used for this analysis of reliability (SPSS Inc., Chicago, IL, USA).

## 3. Results

Considering the Table 1, data analysis showed statistically significant differences in age, height, weight (*p* < 0.0001) except for body mass index (BMI) (*p* = 0.6804). Regarding the Table 2, US measurements for Post 3 deep/crural fascia thickness (*p* < 0.001) were decreased showing statistically significant different for the basketball players group respect the healthy participants group. In addition, the epimysial fascia thickness at Ant 2 level (*p* < 0.01) was increasing reporting statistically significant difference for the basketball players with respect to the healthy participants group. Moreover, no differences were found between the right and left sides in both groups both for deep/crural fascia and epimysial fascia (*p* > 0.05).

### 3.1. Deep/Crural Fascia Ultrasonografic Measurements: Comparison within Each Group

#### 3.1.1. Group A (Basketball Players)

According Bonferroni’s Multiple Comparison Test (Table 3), the comparison between deep/crural fascia thickness among various levels/compartments of the leg showed a statistically significant difference: Ant 2b vs. Post 1b (*p* < 0.001); Ant 2b vs. Post 3b (*p* < 0.05); Post 1b vs. Post 2b (*p* < 0.05).

#### 3.1.2. Group B (Healthy Participants)

According to the Bonferroni’s Multiple Comparison Test (Table 3), the comparison between deep/crural fascia thickness among various levels/compartments of the leg showed a statistically significant difference: Ant 1 vs. Post 3 (*p* < 0.001); Ant 2 vs. Post 1 (*p* < 0.01); Ant 2 vs. Post 3 (*p* < 0.001); Ant 3 vs. Post 3 (*p* < 0.001); Post 2 vs. Post 3 (*p* < 0.001); Post 3 vs. Lat 1 (*p* < 0.001); Post 3 vs. Lat 2 (*p* < 0.001).

#### 3.1.3. Deep/Crural Fascia Ultrasound Measurements Comparison between Groups A and B

According Student’s t-test (Table 3), the comparison of deep/crural fascia thickness between groups A and B showed a statistically significant difference for Post 3 vs. Post 3b (*p* < 0.001).

### 3.2. Correlation Ultrasound Measurements and Descriptive Data

#### 3.2.1. Correlation Deep/Crural Fascia Ultrasound Measurements and Descriptive Data within Groups A and B

According to the correlation analysis (Table 4), there were statistically significant correlations between deep/crural fascia thickness and age, weight, height, BMI; the correlations were significant in according to different compartment/levels (Table 4).

#### 3.2.2. Correlation Epimysial Fascia Ultrasound Measurements and Descriptive Data Within Groups A and B

In addition, as shown in Table 5, there were statistically significant correlations between epimysial fascia ultrasound thickness within the two groups. In group A it was correlated with the weight (*p* = 0.0323), while in group B with the height (*p* = 0.0050).

### 3.3. Correlation Ultrasound Measurements and Functional Data within Group A

#### 3.3.1. Correlation Deep/Crural Fascia Thickness and Functional Data within Group A

In addition, as shown in Table 6, there were statistically significant correlations between Y balance and deep fascia thickness in some compartment/levels; moreover, between ROM and deep fascia thickness ultrasound measurement.

#### 3.3.2. Correlation Epimysial Fascia Thickness and Functional Data within Group A

According to the correlation analysis (Table 7), there was a statistically correlation between epimysial fascia and ROM (*p* = 0.2298) and Y balance composite score (*p* = 0.0475), respectively.

### 3.4. Intra-Rater Reliability

In addition, the intra-rater reliability reported good reliability. The results were: for epimysial fascia: group A, basket (ICC_3,k_: 0.84; 0.71–0.92), group B, healthy (ICC_3,k_:0.82; 0.67–0.91). Deep/Crural fascia: group A, basket (ICC_3,k_: 0.83; 0.69–0.89), group B, healthy (ICC_3,k_:0.83; 0.73–0.91).

## 4. Discussion

To the current knowledge, this study may be stated as the first study detailing the crural fascia US thickness at different compartments and levels, and the epimysial fascia US thickness in level at the point of best visualization in basketball players with recurrent ankle sprains compared with healthy individuals.

The crural/deep fascia was easily visualized in all analyzed compartments/levels, appearing as linear, hyperchogenic layers. In contrast, it was only possible to evaluate the epimysial fascia of the tibialis anterior muscle at Ant 2 level.

Our findings are consistent with those of Wilke et al. [4], study analysis showed that the posterior compartment was thicker than anterior compartment, probably due to a postural reason. The results revealed statistically significant difference between two groups only for Post 3 level, when the basketball players have a thinner deep fascia respect to healthy group. This alteration resulted also more evident if we considered that the evaluated area was close to the superior ankle retinaculum; the latter is a physiological thickening of the deep fascia around the ankle that helps the joint stability and proprioception, as already demonstrated by Stecco et al. [40]. The decrease in the thickness of the deep/crural fascia in the basketball players at this level could determine different biomechanical action on the retinacula of the ankle and consequently CAI with risk of recurrent ankle sprains.

Within each group there were statically significant difference in the deep/crural fascia US thickness at various compartments/levels. They tend to be thicker posteriorly and laterally, playing an important role in myofascial force transmission in these compartments [41,42,43]. From a postural point of view, the lateral and posterior compartments of the crural fascia are most affected by the load, due to the bipedal position, and therefore, our findings confirmed this role. Lateral compartments allow asymmetric standing whereas the posterior compartments being tilted forward by gravity are mechanically more stimulated than the others. Moreover, in the comparison among crural fascia thickness in different compartments/levels within the same group, there was a statistically significant difference between all the thickness pairs compared. In the group B there were highly significant differences between anterior and posterior compartments in all levels, and between posterior and lateral in all levels showing a better distribution of thickness in the various compartments. In the group A, the differences were statically significant between anterior and posterior, not at all levels, and they were absent between posterior and lateral compartments, but without being in accordance with the results of the group B and with the results of the study by Pavan et al. [42] that demonstrated that crural fascia is stiffer along medial-lateral direction. Crural fascia well adapts to different physical condition during muscle contraction and local volume variation. The adaptation to muscle shape largely implies the lengthening of the crural fascia in the transversal direction.

These results could explain the recurrent ankle sprains within basketballs players that might be caused by the different distribution of props of thickness to maintain the ankle in a behavior of harmonic stability.

In addition, the epimysial fascia was thicker in the group A vs. B (*p* < 0.01). The epimysial fasciae are specific for each muscle and define their form and volume; they are thin but well-organized collagen layers that are strongly connected with the muscles. Their role is to transmit forces between adjacent synergistic muscular fiber bundles. The arrangement of collagen fibers in the epimysial fascia varies between muscles of different shape and functions, for example, the pennate muscles have a thicker epimysium that forms a sheet-like aponeurosis acting as a wide base of a muscle attachment [44]. Gao et al. [45] demonstrated that epimysium from the tibialis anterior muscle of rats became stiffer with animal age. Exercise training cause an increased stiffness, while disuse has the opposite effect [46]. Pavan et al. [47] showed that the thickening of the epimysium is associated with immobilization and aging. Our results were consistent with this literature, and they can explain a deficit in muscular recruitment in basketball players. Indeed, the epimysial fasciae are crucial for the proper functioning of the muscle; therefore, this thickening in the basketball players could be linked to a poor recruitment of muscle fibers [47]. The increase of epimysial fascia US thickness was the expression of the increase of the load and tension during the patterns of movements during sport training of the basketball players due to the load and the force transmission [48,49].

In this preliminary study, we sought to examine the relationship between the US thickness of deep/crural fascia and of the epimysial fascia with the descriptive data (age, height, weight, and BMI). The crural fascia US thickness showed, in the group A, relationship for the Ant 3 with Age, while in group B the relationships are statically significant with age, height, weight, and BMI (Table 4). This correlation probably could explain that the deep/crural fascia of the basketball players had a premature aging, as if the load and the various patterns of movement had aged the deep/crural fascia making it thicker at the level Ant3. The latter level was within a fascial line of force transmission, used during jumping.

With regard to the relationship of deep/crural fascia and epimysial fascia with dynamic balance findings, we decided to use these to assess the basketball players and to correlate the date with US thicknesses. Y-balance test (YBT) is a common method for clinical assessment of dynamic balance being currently used to assess risk of injury in athletes [27,28]. The YBT composite score and the asymmetries in anterior and posterolateral directions were correlated with deep/crural fascia US thicknesses. Moreover, the epimysial fascia US thickness had a negative correlation with the Y balance composite score. As high tissue thickness implies greater instability, the balance deficit might have caused by a thickened fascia, not in all levels, which does not stabilize the ankle.

The association of higher fascial thickness and impaired flexibility was found between Lat 2 and ROM in inversion with a negative correlation for deep/crural fascia, whereas for the epimysial fascia, a negative correlation was found with ROM in plantar flexion. Possibly, we hypothesize that the negative association of fascial thickness with some directions of movements are due to changes in myofascial structure in the same directions, resulting in a different transmission of forces.

The findings of the present study did not intend to provide a cause or explanation for the ankle sprains in basketball players. The authors suggest that the examination of the fascial layers of the leg could help to carry out a complete diagnosis added to a traditional ankle exploration (e.g., symptomatology, clinical examination, and manual therapy exploration). Moreover, our results showed that the implementation of the fasciae of the leg approach to a load and manual therapy program for the prevention and management of individuals with recurrent ankle sprains in basketball players could be beneficial.

This is the first work, to our knowledge, to examine and compare US thicknesses of the fascial layers of the leg. Future longitudinal studies including larger numbers of patients will be able to contribute to our knowledge of the pathophysiology of different thickness patterns.

The present study was developed in ultrasound B-Mode, not M-Mode or 4-D mode. In addition, color elastography may be useful to assess the fasciae of the leg. The BMI and age of two groups were not homogeneous, reporting differences between groups that may influence the results.

## 5. Conclusions

To conclude, study results confirm that US imaging is an important tool for assessing the fascial layers of the leg, providing an excellent anatomical definition. The posterior compartment of crural fascia was thicker than the anterior compartment, probably due to a postural reason in both groups. Moreover, they showed an increase in thickness of the epimysial fascia in basketball players with previous ankle sprains. The variability found underlines the importance of establishing standardized landmarks to assess the fasciae and to make results comparable.

## Figures and Tables

**Table 1:** Descriptive data of the sample.

Data	Group A	Group B	*p*-Value Group A vs. Group B
Age, y	25.30 ± 4.04	38.07 ± 12.38	*p* < 0.0001
Weight, kg	84.73 ± 7.22	70.60 ± 12.20	*p* < 0.0001
Height, cm	188.9 ± 6.32	171.30 ± 6.76	*p* < 0.0001
BMI, kg/m^2^	23.73 ± 1.19	24.05 ± 4.03	*p* = 0.6804

Abbreviations: BMI, Body Mass Index. Mean ± standard deviation (SD) was applied.

**Table 2:** Ultrasound measurements of crural fascia in both groups.

Data	Group A	Group B	*p*-Value Group A vs. Group B
Thickness, mm of crural fascia			
Ant 1	0.84 ± 0.15	0.89 ± 0.16	*p* > 0.05
Ant 2	0.74 ± 0.11	0.78 ± 0.15	*p* > 0.05
Ant 3	0.82 ± 0.12	0.81 ± 0.18	*p* > 0.05
Lat 1	0.84 ± 0.16	0.94 ± 0.16	*p* > 0.05
Lat 2	0.87 ± 0.20	0.93 ± 0.15	*p* > 0.05
Post 1	0.99 ± 0.19	1.03 ± 0.28	*p* > 0.05
Post 2	0.79 ± 0.15	0.94 ± 0.27	*p* > 0.05
Post 3	0.94 ± 0.26	1.21 ± 0.46	*p* < 0.001
Thickness, mm of epimysial fascia	0.46 ± 0.10	0.35 ± 0.08	*p* < 0.01
Ant 2			

Abbreviations: BMI, Body Mass Index. Mean ± standard deviation (SD) was applied. In bold: the statistically significant *p*-values.

**Table 3:** Ultrasound measurements comparison within each group and between groups A and B. Only statistically significant differences are reported.

Type of Comparison	Mean Diff.	*t*	*p*-Value	95% CI of Diff
Ant 1 vs. Post 3 *	−0.3132	5.817	*p* < 0.001	−0.5047 to −0.1217
Ant 2 vs. Post 1 *	−0.2423	4.501	*p* < 0.01	−0.4338 to −0.05085
Ant 2 vs. Post 3 *	−0.4248	7.891	*p* < 0.001	−0.6163 to −0.2333
Ant 3 vs. Post 3 *	−0.3942	7.322	*p* < 0.001	−0.5857 to −0.2027
Post 2 vs. Post 3 *	−0.2635	4.894	*p* < 0.001	−0.4550 to −0.07201
Post 3 vs. Lat 1 *	0.2648	4.919	*p* < 0.001	0.07335 to 0.4563
Post 3 vs. Lat 2 *	0.2728	5.068	*p* < 0.001	0.08135 to 0.4643
Post 3 vs. Post 3b °	0.2652	4.925	*p* < 0.001	0.07368 to 0.4567
Ant 2b vs. Post 1b *	−0.2517	4.675	*p* < 0.001	−0.4432 to −0.06018
Ant 2b vs. Post 3b *	−0.2030	3.771	*p* < 0.05	−0.3945 to −0.01151
Post 1b vs. Post 2b *	0.2017	3.746	*p* < 0.05	0.01018 to 0.3932

Abbreviations: b = basketball players. * Mean ± standard deviation (SD) was applied. * = Bonferroni’s test for multiple comparisons. ° = Student’s *t*-test.

**Table 4:** Correlation (Pearson R coefficient test) between Deep/crural fascia Ultrasound measurements and descriptive data within groups A and B. Only statistically significant data are reported.

Compartments/Levels	Data	r	*p*-Value	95% CI of Diff
Ant 3 (group A)	Age	0.4018	*p* = 0.0277	0.04846 to 0.6658
Ant 2 (group B)	Age	−0.4193	*p* = 0.0211	−0.6773 to −0.06943
Ant 3 (group B)	Age	−0.3891	*p* = 0.0336	−0.6573 to −0.03339
Post 2 (group B)	Age	0.3619	*p* = 0.0494	0.001834 to 0.6390
Post 3 (group B)	Age	0.3901	*p* = 0.0331	0.03463 to 0.6580
Post 1 (group B)	Height	−0.3648	*p* = 0.0475	−0.6409 to −0.005143
Post 2 (group B)	Height	−0.4418	*p* = 0.0145	−0.6920 to −0.09686
Lat 1 (group B)	Height	0.4813	*p* = 0.0071	0.1463 to 0.7172
Lat 1 (group B)	Weight	0.4301	*p* = 0.0177	0.08258 to 0.6844
Ant 1 (group B)	BMI	0.3865	*p* = 0.0349	0.03044 to 0.6556
Post 2 (group B)	BMI	0.486	*p* = 0.0065	0.1523 to 0.7202
Post 3 (group B)	BMI	0.4287	*p* = 0.0181	0.08084 to 0.6835

Abbreviations: BMI = body mass index.

**Table 5:** Correlation (Pearson R coefficient test) between Epimysial fascia Ultrasound measurements and descriptive data within groups A and B. Only statistically significant data are reported.

Group	Data	r	*p*-Value	95% CI of Diff
A	Weight	0.3917	*p* = 0.0323	0.03662 to 0.6590
B	Height	−0.4987	*p* = 0.0050	−0.7282 to −0.1688

**Table 6:** Correlation (Pearson R coefficient test) between deep/crural fascia Ultrasound measurements and Y balance test (composite score; asymmetry), ROM within group A. Only statistically significant data are reported.

Type of Fascia (Compartment/Level)	Data	r	*p*-Value	95% CI of Diff
Deep fascia (Ant 3)	Y balance c.s.	0.4494	*p* = 0.0213	0.07515 to 0.7127
Deep fascia (Ant 3)	Y b. as. Ant.	0.4766	*p* = 0.0138	0.1094 to 0.7293
Deep fascia (Lat 1)	Y b. as. Ant.	0.5546	*p* = 0.0033	0.2130 to 0.7754
Deep fascia (Lat 2)	Y b. as. P. lat.	0.6014	*p* = 0.0012	0.2790 to 0.8019
Deep fascia (Post 3)	Y b. as. P. lat.	0.4191	*p* = 0.0331	0.03796 to 0.6938
Deep fascia (Lat 2)	ROM Invers.	−0.4547	*p* = 0.2067	−0.7159 to −0.08171

Abbreviations: c.s. = composite score. As. = asymmetry. Y b. = Y balance. ROM Invers. = ROM Inversione.

**Table 7:** Correlation (Pearson R coefficient test) between epimysial fascia Ultrasound measurements and Y balance test (composite score; asymmetry), ROM, within group. Only statistically significant data are reported.

Group	Data	r	*p*-Value	95% CI of Diff
A	ROM (Pl. F.)	−0.4793	*p* = 0.2298	−0.7310 to −0.1130
A	Y b. c.s.	−0.3923	*p* = 0.0475	−0.6768 to −0.0058

Abbreviations: ROM = range of motion. Pl. F. = plantar flexion. Y b. = Y balance. C.s. = composite score.

## Data Availability

The data presented in this study are available on request from the corresponding author. The data are not publicly available due to privacy.
